# Supplementary material for: Glutamic Acid at Position 343 in PB2 Contributes to the Virulence of H1N1 Swine Influenza Virus in Mice
Source: Viruses. 2025 Jul 20;17(7):1018. doi: 10.3390/v17071018 (PMC12298360; doi:10.3390/v17071018)
Supplement: Supplementary file 1 [file viruses-17-01018-s001.zip › viruses-3714863-supplementary.pdf]

Table S1. Sequences of primers used in this study.

Purpose	Primer (5'- 3')	
	Forward	Reverse
Virus-like vRNA amplification for RT step	GGCCGTCATGGTGGCGAATCGTTCGTCACATCTCATCTACCTCC	
qPCR primers for virus-like vRNA	GGCCGTCATGGTGGCGAAT	CCACACCCTTAGGTAACCCAGTAGA
Virus-like cRNA amplification for RT step	GCTAGCTTCAGCTAGGCATCAGTAGAAACAAGGGTG	
qPCR primers for virus-like cRNA	TGCGGCACAGATTGAAAGCAACAGA	GCTAGCTTCAGCTAGGCATC
Virus-like mRNA amplification for RT step	CCAGATCGTTCGAGTCGTTTTTTTTTTTTTTTTTAT	
qPCR primers for virus-like mRNA	TGCGGCACAGATTGAAAGCAACAGA	CCAGATCGTTCGAGTCGT
